# Carbon Quantum Dots Accelerating Surface Charge Transfer of 3D PbBiO_2_I Microspheres with Enhanced Broad Spectrum Photocatalytic Activity—Development and Mechanism Insight

**DOI:** 10.3390/ma16031111

**Published:** 2023-01-27

**Authors:** Ruyu Yan, Xinyi Liu, Haijie Zhang, Meng Ye, Zhenxing Wang, Jianjian Yi, Binxian Gu, Qingsong Hu

**Affiliations:** College of Environmental Science and Engineering, Yangzhou University, 196 West Huayang Road, Yangzhou 225127, China

**Keywords:** PbBiO_2_I microspheres, CQDs, ionic liquid, charge separation, interface

## Abstract

The development of a highly efficient, visible-light responsive catalyst for environment purification has been a long-standing exploit, with obstacles to overcome, including inefficient capture of near-infrared photons, undesirable recombination of photo-generated carriers, and insufficient accessible reaction sites. Hence, novel carbon quantum dots (CQDs) modified PbBiO_2_I photocatalyst were synthesized for the first time through an in-situ ionic liquid-induced method. The bridging function of 1-butyl-3-methylimidazolium iodide ([Bmim]I) guarantees the even dispersion of CQDs around PbBiO_2_I surface, for synchronically overcoming the above drawbacks and markedly promoting the degradation efficiency of organic contaminants: (i) CQDs decoration harness solar photons in the near-infrared region; (ii) particular delocalized conjugated construction of CQDs strength via the utilization of photo-induced carriers; (iii) π–π interactions increase the contact between catalyst and organic molecules. Benefiting from these distinguished features, the optimized CQDs/PbBiO_2_I nanocomposite displays significantly enhanced photocatalytic performance towards the elimination of rhodamine B and ciprofloxacin under visible/near-infrared light irradiation. The spin-trapping ESR analysis demonstrates that CQDs modification can boost the concentration of reactive oxygen species (O_2_^•−^). Combined with radicals trapping tests, valence-band spectra, and Mott–Schottky results, a possible photocatalytic mechanism is proposed. This work establishes a significant milestone in constructing CQDs-modified, bismuth-based catalysts for solar energy conversion applications.

## 1. Introduction

Semiconductor photocatalysis is deemed as a promising technique to purify water which is contaminated by various pollutants, such as dyes, antibiotics, endocrine disruptors, and so on [1,2,3]. Taking full advantage of solar energy has already been proven as a “green” strategy to settle environmental contamination and energy crunch [4,5,6]. For the sake of triggering an effective photocatalytic reaction and achieving a satisfying degradation efficiency, it is essential to boost effective interfacial contact between target organic molecules and reactive species, namely to involve more photo-generated carriers in surface catalysis. Additionally, several studies have shown that the photocatalytic efficiency can be improved via increasing the adsorption capability of photocatalysts towards contaminants [7,8,9].

Among various Bi-based semiconductor photocatalysts, BiOI displays some fascinating advantages, e.g., high chemical inertness, easy preparation, low toxicity, and broad visible-light absorption range [10]. Moreover, it displays some photocatalytic performance in the field of pollutants elimination, CO_2_ conversion, N_2_ reduction, and so forth [11,12,13]. Nevertheless, individual BiOI is subjected to its inherent weakness, such as the poor oxidation capacity caused by the high value of valence band and low charge separation efficiency. Moreover, during the contaminant degradation process, the contaminants’ adsorption and activation capacity over BiOI is still unsatisfactory [14,15]. Therefore, a variety of methods have been adopted to improve the photocatalytic performance of BiOI, e.g., elemental doping, defect regulation, exposed facet control, heterojunctions construction and bismuth-rich strategy [16,17,18,19]. In addition, a part of Bi in the [Bi_2_O_2_]^2+^ layer can be replaced by other main group elements (Pb, Ba, Sr, Ca, etc.) to generate [ABiO_2_]^+^ [20,21,22], and I^−^ and [ABiO_2_]^+^ can be arranged alternately to form ABiO_2_I. The construction of bismuth-based bimetallic oxyiodide can maximize the photocatalytic performance of bismuth oxyiodide under broadband light irradiation. Considering that the radii of Pb^2+^ and Bi^3+^ are very close, the crystalline structure of PbBiO_2_I displays no obvious change by substituting Bi^3+^ with Pb^2+^ [23]. More importantly, PbBiO_2_I displays preferable band structure with a suitable narrow bandgap of 1.9 eV, which is beneficial to the degradation of contaminants. Therefore, PbBiO_2_I displays great potential in the field of environmental purification.

As a key member of the carbon-based nanomaterial family, 0D CQDs have drawn tremendous research attention [24,25]. Particular properties, e.g., high solubility, excellent electron conductivity, and up-conversion performance, endow them with broad applications [26,27,28]. Herein, CQDs have been extensively employed to modify photocatalysts to heighten their optical and electrochemical properties, and eventually promote their photocatalytic performance. In fact, the introduction of CQDs can boost the separation and transportation of photo-generated electron-hole pairs and enable more reactive oxygen species participating in the degradation of contaminants [29]. In spite of this, in previous references, the core character of CQDs during the photocatalytic reactions and pollutant degradation mechanism have not yet been studied at length. More importantly, considering that the diameter of CQDs is less than 10 nm, the uniform distribution of CQDs around catalyst surface requires further study.

In order to deeply analyze the aforementioned impending issues, CQDs modified PbBiO_2_I nanocomposite photocatalysts are obtained via an ionic liquid [Bmim]I assisted solvothermal method. In this approach, [Bmim]I can be employed as template and reaction source to control the growth of PbBiO_2_I crystals. In fact, the existence of coulomb force and hydrogen bond between [Bmim]I and CQDs is beneficial to in-situ anchoring more CQDs around PbBiO_2_I material [30]. Moreover, CQDs decoration can promote organic pollutants adsorption, boost interface charge separation and transportation, and ultimately enhance the photocatalytic degradation efficiency of rhodamine B (RhB) and ciprofloxacin (CIP) under visible/near-infrared light irradiation. Our research extends the knowledge into developing more CQDs-decorated, bismuth-based bimetallic catalysts with widespread applications in the area of wastewater treatment.

## 2. Experimental Details

### 2.1. Sample Preparation

CQDs powder was acquired on the basis of the previous reference and then managed by lyophilization [29]. Hence, 1.06 g citric acid monohydrate was dispersed into deionized water (11 mL), and ethylenediamine (340 μL) was injected and stirred for 1 h. This above clear solution was sealed in Teflon-lined autoclave (25 mL) and reacted at 200 °C for 5 h. After cooling down to room temperature, the brownish red solution was subjected to dialysis for 72 h to obtain the CQDs solution. In the end, CQDs powder was obtained after freeze-drying for 72 h.

**Scheme 1:**
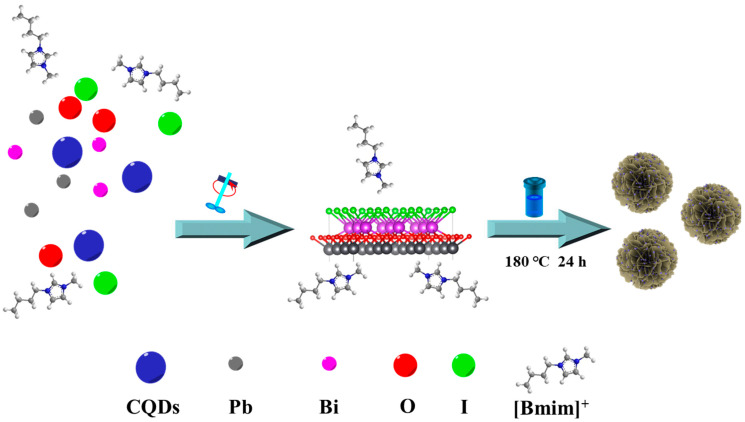
Schematic diagram for the formation of CQDs/PbBiO_2_I nanocomposite catalyst.

The synthetic process of pure PbBiO_2_I and CQDs/PbBiO_2_I nanocomposite was as follows (Scheme 1) via ionothermal method: First of all, CQDs (× *g*), Bi(NO_3_)_3_·5H_2_O (0.24 g) and Pb(NO_3_)_2_ (0.16 g) are fully dispersible in ethylene glycol (15 mL) and defined as A. Then, ionic liquid [Bmim]I (0.13 g) was dispersed uniformly into ethylene glycol (5 mL) and defined as B. B was added into A bit by bit and stirred for 1 h. After that, the mixture was sealed in a Teflon-lined autoclave (25 mL) and reacted at 180 °C for 24 h. Subsequently, the sediment was collected via high-speed centrifugation, rinsed three times with deionized water and absolute ethanol, and dried at 80 °C for 12 h. The loading amount of CQDs in CQDs/PbBiO_2_I nanocomposite was 1, 3, 5, and 8 wt.%, respectively. Individual PbBiO_2_I was also obtained without the introduction of CQDs.

### 2.2. Sample Characterization

X-ray diffraction (XRD) was recorded on a D8 Advance diffractometer (Bruker, Germany) using monochromatic Cu Kα radiation. X-ray photoelectron spectroscopy (XPS) spectrum was measured using an ESCALAB 250Xi XPS (Thermo Fisher, Waltham, MA, USA) with monochromatic Mg-Kα radiation as X-ray source for excitation. A laser Raman spectrometer (DXR, Thermo Fisher Scientific, Waltham, MA, USA) was employed to collect Raman spectra with a 532 nm laser as an excitation source. A specific surface and aperture analyzer (Quadrasorb EVO, Anton Paar, Ashland, VA, USA) was used to analyze the specific surface area and pore diameter of the catalysts by N_2_ adsorption-desorption isotherms analyzed at 77 K using the Brunauer–Emmett–Teller (BET) method and Barret–Joyner–Halenda (BJH) adsorption dV/dW pore volume distribution. The microstructures of the catalysts were investigated by Tecnai G2 F30 S-TWIN transmission electron microscopy (TEM, FEI, USA). UV-Vis diffuse reflection spectra was acquired on a UV-2450 spectrophotometer (Shimadzu, Japan). The photoluminescence (PL) spectra were obtained by a FLS980 fluorescence spectrometer (Edinburgh, UK). An electron spin resonance (ESR) spectrometer (JES-FA200, Bruker, Germany) was used to capture ESR signals of spin-trapped radicals using 5,5-dimethyl-1-pyrroline-*N*-oxide (DMPO, radical trapping reagent) in water and methanol solutions. The electrochemical data were obtained on a CHI 660B electrochemistry workstation (Chenhua, Shanghai) employing a conventional three-electrode cell (ITO slice, Pt wire and saturated Ag/AgCl).

### 2.3. Photocatalytic Degradation Test

RhB and CIP were selected as the model contaminants. A 250 W xenon plus equipped with optical filter (λ > 400 nm or λ > 610 nm) was employed as the optical source. The reaction temperature was kept at 25 °C via a recirculating cooling water system. Hence, 0.03 g samples were added in RhB solution (100 mL, 20 mg/L) or CIP solution (100 mL, 10 mg/L) by ultrasonic dispersion. The mixed solution was stirred in the dark for 30 min. Subsequently, the optical source was switched on, and 4.5 mL reaction mixture was sampled at set intervals. The acquired mixture was centrifuged at 15,000 rpm for 4 min to acquire a clear solution. The concentrations of RhB and CIP were studied using a Shimadzu LC-20A HPLC system (Shimadzu, Japan), including an Agilent TC-C (18) column, two Varian Prostar 210 pumps and a ultraviolet detector. A mobile phase consisting of methanol and pure water in the ratio of 30:70 (*v*:*v*) was used at the flow rate of 0.8 mL min^−1^. The reaction solution (10 μL) was injected. To distinguish the major reactive species participating in photocatalytic reactions, various radical scavengers were introduced. Tert-butanol (t-butanol) can capture hydroxyl radical (•OH), nitrogen (N_2_) can inhibit the production of superoxide radical (O_2_^•−^), while ammonium oxalate (AO) and triethanolamine (TEA) can capture holes (h^+^).

## 3. Result and Discussion

**Figure 1:**
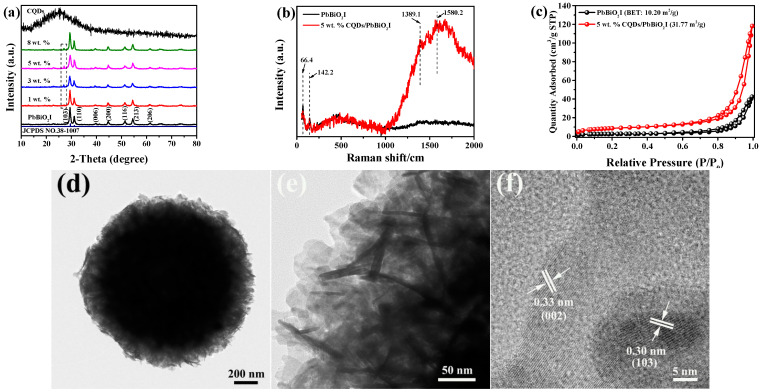
(**a**) XRD patterns of the as-prepared samples; (**b**) Raman spectra and (**c**) Nitrogen adsorption-desorption isotherms of PbBiO_2_I and 5 wt.% CQDs/PbBiO_2_I; (**d**,**e**) TEM and (**f**) HRTEM images of 5 wt.% CQDs/PbBiO_2_I.

The crystal structures of the acquired materials were analyzed by the X-ray diffraction patterns (XRD). As depicted in Figure 1a, all the peaks can be indexed to tetragonal PbBiO_2_I (JCPDS NO. 38-1007) [31]. For single CQDs, a broad peak located at 25° can be observed. After the decoration of CQDs, the nanocomposite retains the same diffraction peaks of tetragonal PbBiO_2_I. Additionally, it is noteworthy that a small peak centered around 26.9° appears with the increased loading amount of CQDs. This phenomenon validates the successful introduction of CQDs. Raman spectra was conducted to further confirm the successful loading of CQDs. As shown in Figure 1b, the strong peaks (66.4 and 142.2 cm^−1^) are ascribed to the “lattice vibration” of PbBiO_2_I. Compared with single PbBiO_2_I, 5 wt.% CQDs/PbBiO_2_I displays two apparent peaks centered at 1389.1 and 1580.2 cm^−1^, which represent the disordered (D) and graphitic (G) bands of graphene. The peak intensity of G band is much higher than that of D band, which is in agreement with previous report [32]. There is background interference in the nanocomposite because of the strong fluorescence for CQDs. To explore the textural properties of the obtained samples, BET surface area measurement was carried out. In Figure 1c, the N_2_ adsorption/desorption isotherms can be categorized as type IV isotherm, indicating the characteristics of mesoporous [33]. This is in accord with the pore diameter distribution analysis (Figure S1). Moreover, the BET value of PbBiO_2_I and 5 wt.% CQDs/PbBiO_2_I is determined to be 10.20 and 31.77 m^2^ g^−1^. As is well known to all, a higher surface area is beneficial to provide more exposed reactive sites and transport paths for reactants and products, probably resulting in the enhanced photocatalytic performance [34].

The morphology of CQDs and CQDs/PbBiO_2_I was studied by TEM analysis. As shown in Figure S2, the CQDs are monodisperse with a spherical shape diameter of 5 nm. Moreover, the lattice fringe spacing of 0.33 nm corresponds to the (002) plane of CQDs [35]. For CQDs/PbBiO_2_I nanocomposite, it displays flower-like spheres with a diameter of 2 μm assembled from nanosheets (Figure 1d,e). To further observe this microstructure, high-resolution TEM (HRTEM) measurement was carried out. As shown in Figure 1f, the lattice fringe spacing of 0.33 nm and 0.30 nm corresponds to the (002) plane of CQDs and (103) crystallographic plane of PbBiO_2_I. The results of TEM analysis demonstrate that CQDs/PbBiO_2_I nanocomposite catalyst have been constructed successfully.

**Figure 2:**
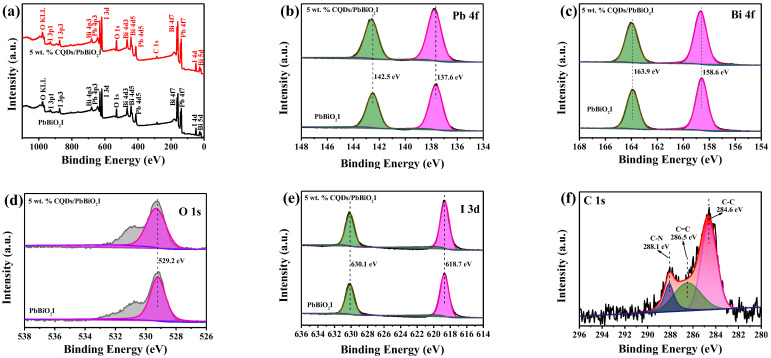
XPS spectra of PbBiO_2_I and 5 wt.% CQDs/PbBiO_2_I: (**a**) survey, (**b**) Pb 4f, (**c**) Bi 4f, (**d**) O 1s, (**e**) I 3d, (**f**) C 1s.

The surface chemical states and compositions of the obtained catalysts were investigated by X-ray photoelectron (XPS) spectra (Figure 2). The survey XPS spectrum shows the co-existence of Pb, Bi, O, I, and C elements in the CQDs/PbBiO_2_I nanocomposite (Figure 2a). In Figure 2b, the peaks at roughly 142.5 and 137.6 eV are ascribed to the Pb 4f of Pb^2+^ [35]. In Figure 2c, the strong peaks located at 163.9 and 158.6 eV are assigned to the Bi 4f_5/2_ and Bi 4f_7/2_, manifesting the presence of trivalent bismuth [36]. What is more, after the decoration of CQDs, the binding energies of Pb 4f and Bi 4f shift to higher values when compared to those of PbBiO_2_I. The binding energy shift unequivocally proves the occurrence of electron re-distribution between PbBiO_2_I and CQDs. Similar phenomena have been reported in other references [29,37]. The binding energy of O 1s centered at 529.2 eV (Figure 2d) is assigned to the oxygen of PbBiO_2_I. The XPS peaks of I 3d centered at 630.1 and 618.7 eV are ascribed to I 3d_3/2_ and I 3d_5/2_ of I^−^ [31] (Figure 2e). Figure 2f displays the high-resolution XPS spectra of C 1s, in which three deconvoluted peaks can be ascribed to C-C (284.6 eV), C=C (286.5 eV) and C-N (288.1 eV), respectively [26,29]. The XPS results further prove the presence of PbBiO_2_I and CQDs in the CQDs/PbBiO_2_I nanocomposite.

**Figure 3:**
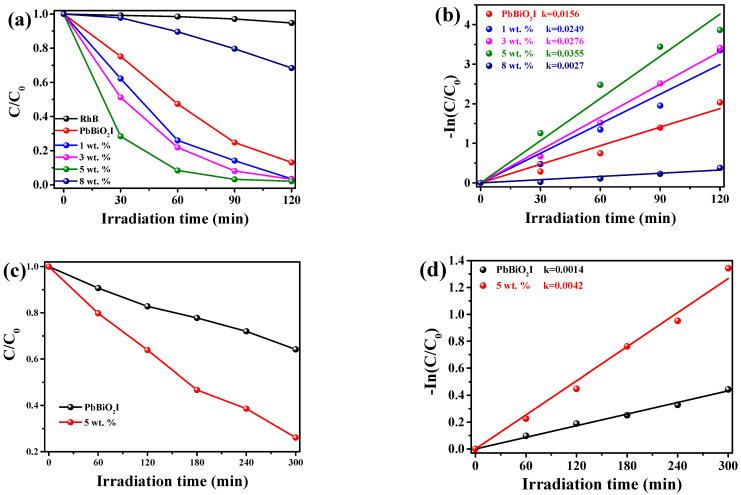
Photocatalytic properties of the catalysts for the degradation of RhB (**a**) and CIP (**c**) under visible light irradiation (λ > 400 nm); reaction kinetics for the degradation of RhB (**b**) and CIP (**d**) under the same condition.

The photoreactivity of the as-prepared catalysts was assessed by decomposition of RhB upon irradiation with visible light (λ > 400 nm). As we all know, RhB is broadly used as a coloration in textile and food processing industries and is also a popular water tracer fluorescent. It poses a threat to human beings and animals, and causes irritation of the eyes, skin, and respiratory passage. The carcinogenicity, reproductive and development toxicity and chronic toxicity toward human beings and animals have been proven experimentally [38]. Therefore, it is crucial to reduce RhB concentrations to reach the national standards. Herein, CQDs/PbBiO_2_I nanocomposite are tentatively employed to remove RhB, and the importance of CQDs can also be confirmed from another angle. The RhB adsorption capacity over different samples is shown in Figure S3. The dark adsorption experiment results demonstrate that the adsorption capacity can be improved thanks to the π-π interactions between CQDs and RhB molecule [36]. In Figure 3a, the photolysis of RhB is almost negligible. For PbBiO_2_I, the degradation rate of RhB is only 29.7% within 30 min. After the introduction of CQDs, CQDs/PbBiO_2_I nanocomposites show higher photocatalytic efficiency than that of PbBiO_2_I, highlighting the key role of CQDs in environmental purification. Notably, 5 wt.% CQDs/PbBiO_2_I is obviously superior to other CQDs modified PbBiO_2_I samples. This can be explained as too many CQDs may generate an adverse shielding effect, hindering the PbBiO_2_I surface from absorbing visible-light photons and overlaying the reactive sites for photocatalysis via CQDs agglomeration [36,39]. Figure 3b shows the photocatalytic kinetics fit of RhB degradation on account of pseudo-first-order model (Langmuir-Hinshelwood model). It can be clearly seen that the degradation efficiency of RhB can be obviously enhanced after adding CQDs. Specifically, the degradation rate of 5 wt.% CQDs/PbBiO_2_I is approximately 2.27 times larger than that of PbBiO_2_I. To evaluate the catalyst’s reusability, five consecutive cycles were conducted over 5 wt.% CQDs/PbBiO_2_I photocatalyst. In Figure S4a, the degradation rate remains 90% in the elimination of RhB, manifesting the preferable catalytic stability of the nanocomposite catalyst. Furthermore, XRD patterns further confirm the well-retained structure of the nanocomposite catalyst after photoirradiation reaction (Figure S4b).

CIP is classified as belonging the second generation of fluoroquinolone antibiotics, being proven to damage the environment and display toxic effects in the surface water and groundwater [40]. This is the first report employing CQDs/PbBiO_2_I nanocomposite for CIP degradation, and the degradation plots are shown in Figure 3c. The degradation rate of CIP achieves 35.8% within 300 min while employing PbBiO_2_I as catalyst. Surprisingly, more than 73.9% of CIP can be eliminated over PbBiO_2_I loading with 5 wt.% CQDs. Further, the calculating rate constant is 0.0042 min^−1^, which is much higher than that of PbBiO_2_I (0.0014 min^−1^) (Figure 3d). Additionally, a total organic carbon (TOC) experiment was carried out to study the mineralization of RhB and CIP over 5 wt.% CQDs/PbBiO_2_I (Figure S5). Under visible-light irradiation for 120 min, almost 76.2% of RhB is mineralized. For CIP, approximately 40.1% of CIP can be mineralized under illumination for 300 min. This implies that both RhB and CIP can be mineralized effectively over 5 wt.% CQDs/PbBiO_2_I under visible light irradiation.

**Figure 4:**
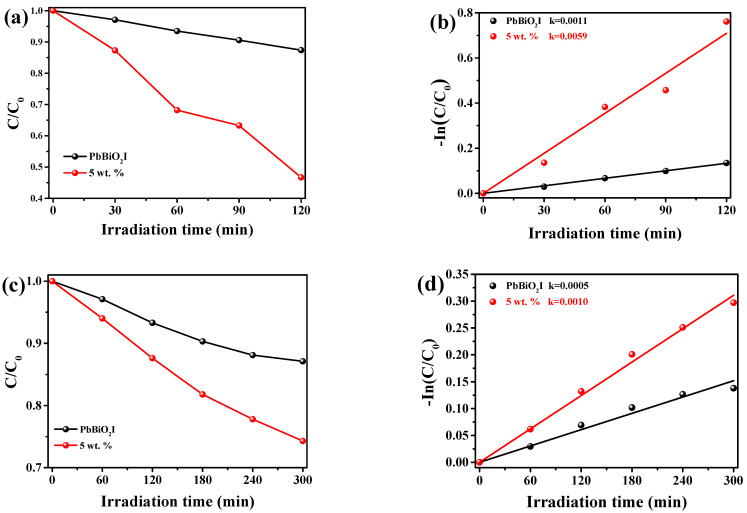
Photocatalytic properties of the catalysts for the degradation of RhB (**a**) and CIP (**c**) under near-infrared light irradiation (λ > 610 nm); reaction kinetics for the degradation of RhB (**b**) and CIP (**d**) under the same condition.

The photocatalytic performance of single PbBiO_2_I and 5 wt.% CQDs/PbBiO_2_I was further studied under near-infrared photoirradiation (λ > 610 nm). As shown in Figure 4a, only 13.5% RhB can be degraded by PbBiO_2_I after 120 min near-infrared photoirradiation. Accompanied with the decoration of CQDs, the photocatalytic efficiency is significantly improved and 53.3% RhB is degraded over 5 wt.% CQDs/PbBiO_2_I under the same condition. The corresponding rate constant for 5 wt.% CQDs/PbBiO_2_I is 5.36 times higher than pure PbBiO_2_I (Figure 4b). The ratio of rate constant for 5 wt.% CQDs/PbBiO_2_I to individual PbBiO_2_I under near-infrared photoirradiation is analogous to the ratio under visible light irradiation, which indicate the analogical activation pattern. In comparison to visible light condition, the ratio of rate constant for 5 wt.% CQDs/PbBiO_2_I to individual PbBiO_2_I under near-infrared light condition is higher, indicating that CQDs can transport photo-generated electrons more efficiently under near-infrared photoirradiation [29]. Given that the dye-sensitization effect involved in the degradation of RhB, the degradation of colorless CIP was carried out under the same condition, pure PbBiO_2_I only degrades 12.9% CIP after 300 min irradiation. After the modification of CQDs, 5 wt.% CQDs/PbBiO_2_I can degrade 25.7% CIP after 300 min irradiation (Figure 4c). The reaction rate constant of 5 wt.% CQDs/PbBiO_2_I is 2.0 times that of PbBiO_2_I (Figure 4d). The above experimental data demonstrate the key roles of CQDs during a photocatalytic reaction process [29,41].

**Figure 5:**
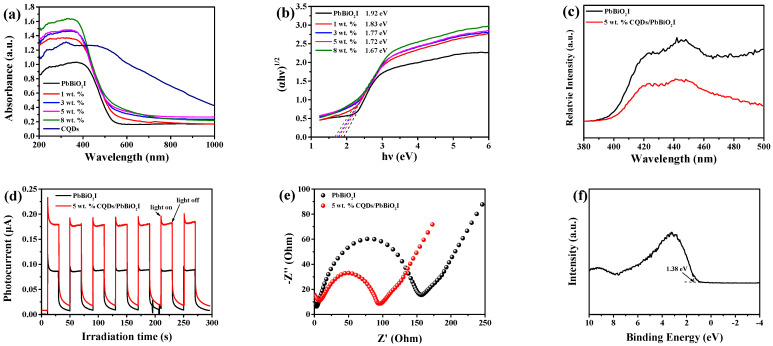
(**a**) UV-vis DRS of the acquired samples; (**b**) Bandgap of PbBiO_2_I and CQDs/PbBiO_2_I nanocomposite; (**c**) PL spectra, (**d**) transient photocurrent spectra, (**e**) EIS spectra of PbBiO_2_I and 5 wt.% CQDs/PbBiO_2_I; (**f**) XPS Valence-band spectra of PbBiO_2_I.

The optical characteristics of the obtained samples across the UV-Vis region are recorded by diffuse reflectance spectra (DRS). In Figure 5a, pristine PbBiO_2_I displays intrinsic bandgap absorption from 200 nm to 570 nm. This can be attributed to their intrinsic band-to-band transition [36]. After the modification of CQDs, the optical absorption is substantially extended to the near-infrared region. As a result, it may boost the generation of photo-induced carriers thanks to the enhanced light-harvesting capability. Consequently, more reactive species can be involved in the photocatalytic reaction. The E_g_ values of PbBiO_2_I and CQDs/PbBiO_2_I nanocomposite can be obtained employing the Kubelka–Munk function [8]:

αhν = A (hν − E_g_)^n/2^

where α, h, ν, A, and E_g_ represent the absorption coefficient, Planck constant, light frequency, a constant, and band gap energy, respectively. As depicted in Figure 5b, the E_g_ values of PbBiO_2_I, 1 wt.% CQDs/PbBiO_2_I, 3 wt.% CQDs/PbBiO_2_I, 5 wt.% CQDs/PbBiO_2_I, and 8 wt.% CQDs/PbBiO_2_I are calculated to be 1.92, 1.83, 1.77, 1.72, and 1.67 eV, respectively.

To clarify the charge separation and transfer kinetics over the obtained catalysts, photoluminescence (PL) and photoelectrochemical measurements are performed. Figure 5c displays that both PbBiO_2_I and 5 wt.% CQDs/PbBiO_2_I possess an obvious emission peak centered at 440 nm. More importantly, 5 wt.% CQDs/PbBiO_2_I shows an apparent quenching in comparison to PbBiO_2_I. This characterization result indicates an improving separation probability of photo-generated carriers, which is beneficial for visible/near-infrared light-driven catalytic reactions [42]. The charge separation/transfer process is further monitored by transient photocurrent and electrochemical impedance spectroscopy (EIS) measurements. Figure 5d exhibits the transient photocurrent responses of the two samples under chopped light irradiation. It can be found that the photocurrent density is in the order PbBiO_2_I < 5 wt.% CQDs/PbBiO_2_I, which coincide with the trend of photocatalytic performance. The photocurrent results demonstrate that the charge separation efficiency of 5 wt.% CQDs/PbBiO_2_I is superior to that of PbBiO_2_I [43]. The results of transient photocurrent are further confirmed by EIS. In Figure 5e, the semicircle arc of 5 wt.% CQDs/PbBiO_2_I in the Nyquist plot is smaller than that of PbBiO_2_I, reflecting the lower interfacial charge transfer resistance [44]. Taking the above PL and photoelectrochemical results into account, CQDs modification will boost charge migration and separation, which is favorable for the generation of reactive species.

Apart from the light absorption efficiency and spatial separation efficiency of photoinduced charge carriers, the energy band structure also plays a critical role in determining photocatalytic efficiency. The total density of states of valence band (VB) that can be obtained based on the valence-band XPS spectra with Fermi level (*E*_f_) of semiconductors is 0 eV (Figure 5f). The VB value of PbBiO_2_I is measured to be 1.38 eV, and the positive slopes of Mott–Schottky curves show that PbBiO_2_I is defined as a n-type semiconductor (Figure S6). According to the extrapolation of *X* intercept in the Mott–Schottky plots, the flat band potential of PbBiO_2_I is measured to be −0.62 V vs. NHE (pH = 7). With regarding to the n-type semiconductors, the Fermi level is close to the flat band potential [45]. As a result, the VB value of PbBiO_2_I is 0.76 V vs. NHE. On the basis of the *E*_g_, the CB minimum of PbBiO_2_I occur at approximately −1.16 V vs. NHE.

**Figure 6:**
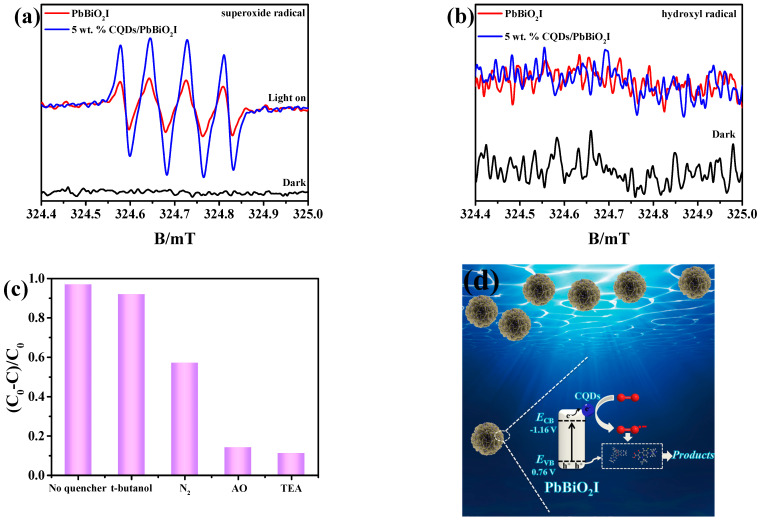
(**a**) DMPO- O_2_^•−^ and (**b**) DMPO-•OH of PbBiO_2_I and 5 wt.% CQDs/PbBiO_2_I; (**c**) trapping experiments of active species during the degradation of RhB; (**d**) photocatalytic mechanism of CQDs/PbBiO_2_I nanocomposite in photocatalytic degradation.

The ESR (electron spin resonance) technique and free radicals capturing tests are conducted to ascertain the major active species involved in the degradation process [46,47]. The results of ESR analysis are presented in Figure 6a,b. In the darkness, no characteristic signals can be observed for DMPO-O_2_^•−^ and DMPO-•OH from the two catalysts, and no reactive species can be trapped. Nevertheless, typical characteristic signal peaks of DMPO-O_2_^•−^ are observed under photoirradiation, and the DMPO-O_2_^•−^ signals of 5 wt.% CQDs/PbBiO_2_I are obviously higher than that of PbBiO_2_I. Furthermore, DMPO-•OH cannot be trapped from the two catalysts upon light illumination. ESR analysis demonstrate that superoxide radical (O_2_^•−^) can be generated upon light illumination, and the modification of CQDs is beneficial for the generation of reactive oxygen species. To further verify the presence of these active species, free radical quenching tests are carried out in the presence of 5 wt.% CQDs/PbBiO_2_I (Figure 6c). After the addition of AO and TEA, notable inhibition of photocatalytic activity can be observed, indicating that direct hole oxidation plays a critical role during visible/near-infrared light-driven catalytic reactions. Moreover, after N_2_ is pumped into the reaction solution, the degradation efficiency declines greatly [48]. This can be explained as a large amount of O_2_^•−^ is being generated and acting in a key role during the degradation process. The analysis of radical capture tests are in accordance with ESR results.

Considering the above experimental results, the separation and transformation paths of photo-generated carriers involved in visible/near-infrared light-driven degradation of organic contaminants are presented in Figure 6d. Under broadband light irradiation, electrons in the VB of PbBiO_2_I can be motivated and then migrated to the CB. Because of the narrow band gap width, the recombination of photo-induced electrons and holes occurs in very little time. After the modification of CQDs, the mobility of photo-generated electrons can be enhanced thanks to the electron acceptor property of CQDs. Consequently, more powerful oxidants participate in photocatalytic reactions. Even so, the holes on the VB cannot thermodynamically oxidize H_2_O (H_2_O/•OH 2.34 V vs. NHE) or OH^−^ (•OH/OH^−^ 1.99 V vs. NHE) to generate •OH [37,49]. Therefore, the main reactive species, including O_2_^•−^ and holes generated under visible/near-infrared light illumination, engage in organic contaminant elimination together, synergistically boosting the enhancement of photocatalytic performance over the CQDs/PbBiO_2_I nanocomposite.

## 4. Conclusions

In conclusion, CQDs modified PbBiO_2_I microspheres have been fabricated via a simple ionothermal method. (i) In this prepared procedure, ionic liquid serves as a high performance template, reactant, and dispersant, which is beneficial for the uniform distribution of CQDs around PbBiO_2_I microspheres; (ii) the photocatalytic activity for removing organic contaminants is greatly enhanced; and (iii) CQDs modification can successfully reduce charge carrier recombination and accelerate the transformation of photoinduced hole-electron pairs to the catalyst surface. Therefore, more reactive oxygen species can be generated and involved in the elimination of RhB and CIP. This work may offer some insights for constructing a carbon-based material-modified, bismuth-based catalyst, which will be widely welcomed in environmental purification and energy conversion.

## Data Availability

The data presented in this study are available upon request from the corresponding author.

## Supplementary Materials

The following Supplementary Materials can be downloaded at: https://www.mdpi.com/article/10.3390/ma16031111/s1. Figure S1: The pore size distribution curves of PbBiO_2_I and 5 wt.% CQDs/PbBiO_2_I; Figure S2: HR-TEM image of CQDs; Figure S3: The adsorption equilibrium of RhB over various catalysts in the darkness; Figure S4: Cycling runs for RhB degradation over 5 wt.% CQDs/PbBiO_2_I under visible light irradiation (λ > 400 nm) (a), XRD patterns of 5 wt.% CQDs/PbBiO_2_I before and after five cycles (b); Figure S5: The decrease of TOC during the photodegradation of RhB (a) and CIP (b) over 5 wt.% CQDs/PbBiO_2_I; Figure S6: Mott-Schottky plots of PbBiO_2_I.
