# Supplementary material for: Carbon Quantum Dots Accelerating Surface Charge Transfer of 3D PbBiO2I Microspheres with Enhanced Broad Spectrum Photocatalytic Activity—Development and Mechanism Insight
Source: Materials (Basel). 2023 Jan 27;16(3):1111. doi: 10.3390/ma16031111 (PMC9918922; doi:10.3390/ma16031111)
Supplement: Supplementary file 1 [file materials-16-01111-s001.zip › materials-2104050-supplementary.pdf]

## Supplementary Materials

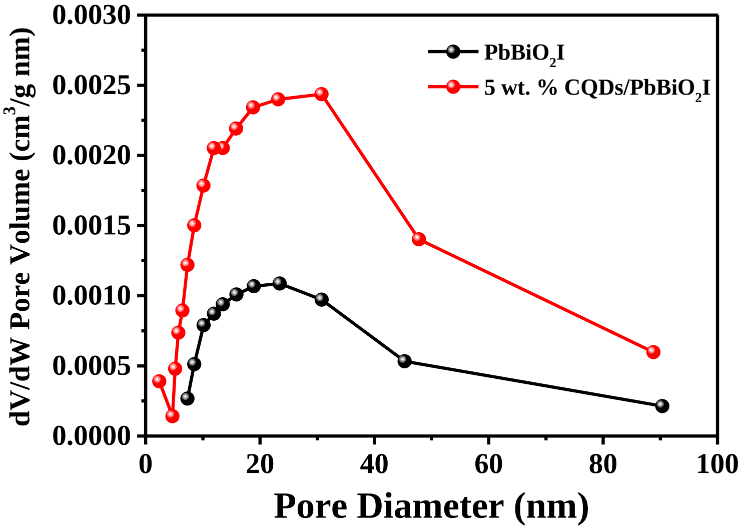


**Figure S1.** The pore size distribution curves of $PbBiO_2I$ and 5 wt. % CQDs/$PbBiO_2I$.

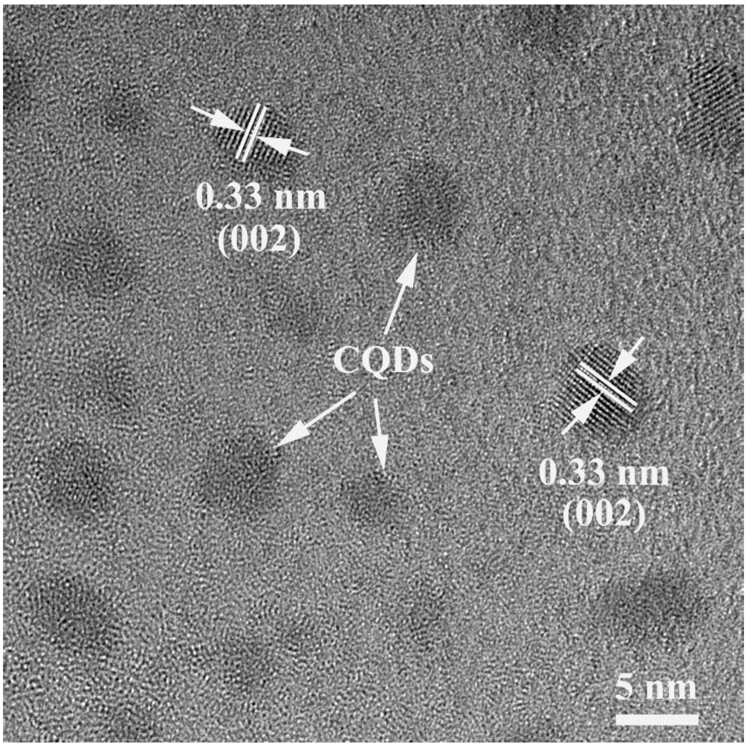


**Figure S2.** HR-TEM image of CQDs.

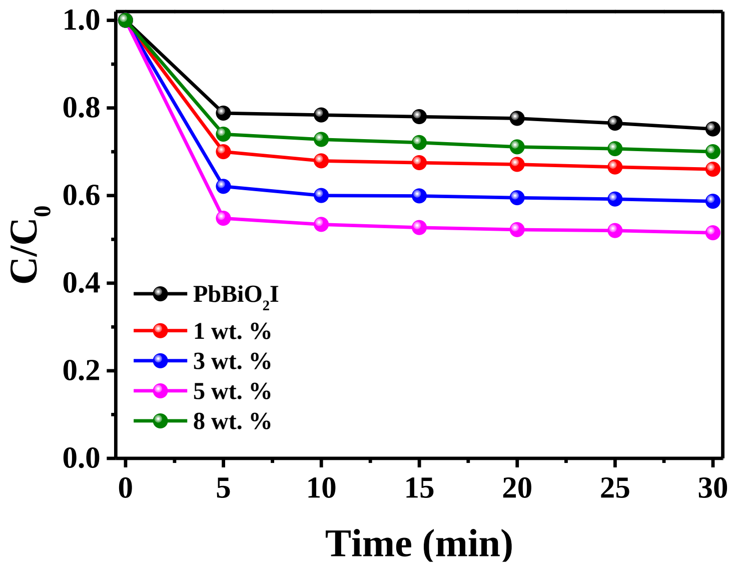


**Figure S3.** The adsorption equilibrium of RhB over various catalysts in the darkness.

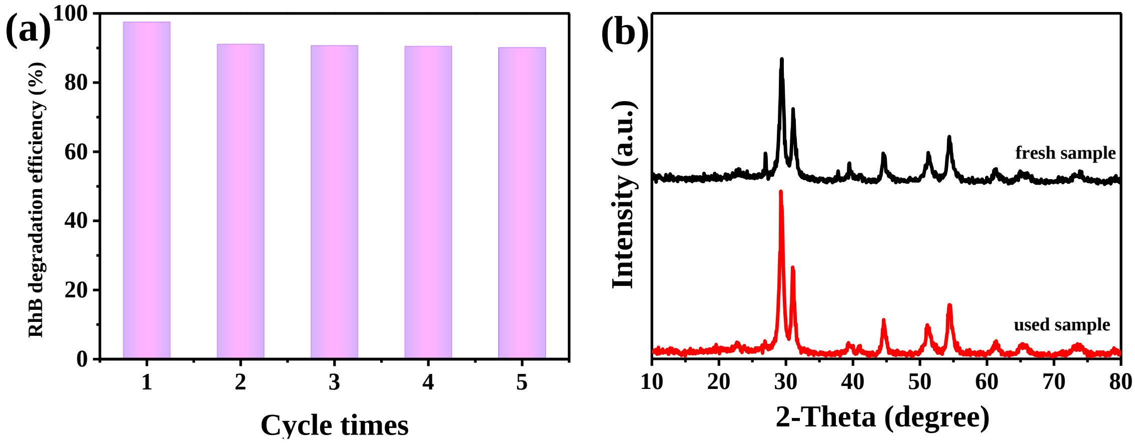


**Figure S4**. Cycling runs for RhB degradation over 5 wt. % CQDs/$PbBiO_2I$ under visible light irradiation ($\lambda > 400$ nm) (**a**), XRD patterns of 5 wt. % CQDs/$PbBiO_2I$ before and after five cycles (**b**).

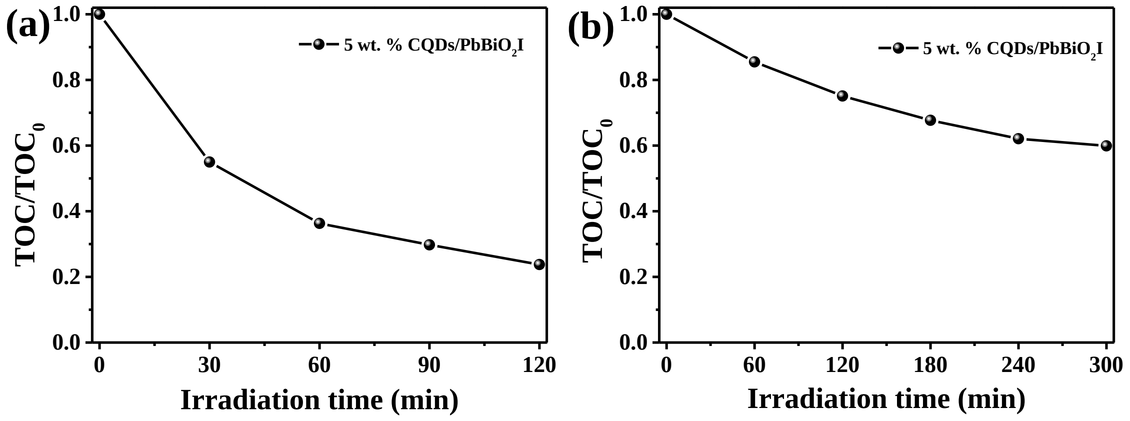


**Figure S5.** The decrease of TOC during the photodegradation of RhB (a) and CIP (b) over 5 wt. % CQDs/PbBiO$_2$I.

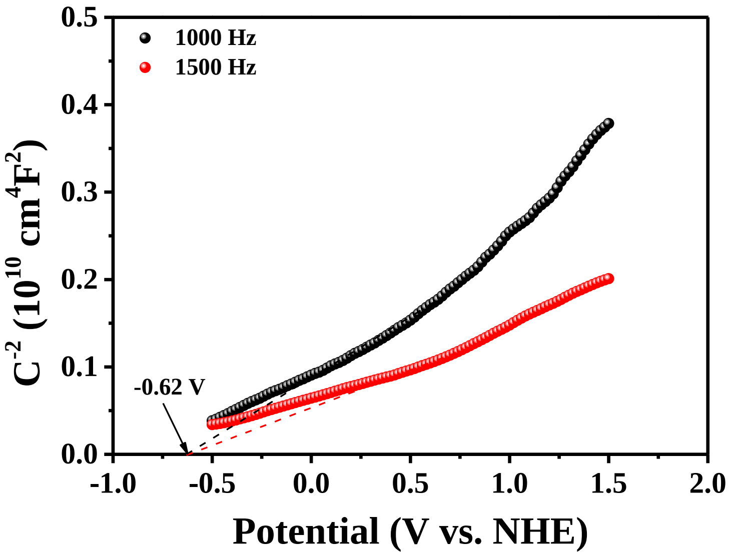


**Figure S6**. Mott-Schottky plots of PbBiO$_2$I.
